# Dilemma in the Treatment of Sports Injuries in Athletes: Tendon Overuse, Muscle Strain, and Tendon Rupture

**DOI:** 10.1111/sms.70026

**Published:** 2025-02-20

**Authors:** Michael Kjær, Jesper Petersen, Michael Ries Dünweber, Jesper Løvind Andersen, Lars Engebretsen, Stig Peter Magnusson

**Affiliations:** ^1^ Department of Orthopaedic Surgery, Institute of Sports Medicine Copenhagen Copenhagen University Hospital—Bispebjerg and Frederiksberg Copenhagen Denmark; ^2^ Department of Clinical Medicine, Faculty of Health and Medical Sciences University of Copenhagen Copenhagen Denmark; ^3^ Team Danmark Brøndby Denmark; ^4^ Department of Orthopaedic Surgery, Norwegian School of Sport Sciences University of Oslo Oslo Norway; ^5^ Oslo Sports Trauma Research Center Oslo Norway; ^6^ Department of Physical and Occupational Therapy Copenhagen University Hospital—Bispebjerg and Frederiksberg Copenhagen Denmark

**Keywords:** Achilles tendon rupture, muscle strain, tendinopathy

## Abstract

Injuries to the musculoskeletal system are frequent in elite sports and they are detrimental to athletic performance. This can be due to, for example, (1) overuse disorders of tendon (tendinopathy) that not only lower the training efficiency but also, in many cases, are career‐ending for the athlete due to pain; (2) acute muscle strain injury that both causes prolonged absence from competition and results in many re‐injuries; or (3) tendon rupture that, apart from a very long rehabilitation period, will also result in many athletes never resuming their original high level of competitive sports. For all three injury examples, several evidence‐based prevention programs and treatments are available, and yet the incidence of these injuries remains high and single treatments often provide only partial recovery. In this paper, we highlight the current treatments of these three conditions and focus on the unsolved dilemmas that exist in these sports injuries.

## 1 Tendon Overuse Injury (Tendinopathy)

Overuse injuries of tendons are a major challenge to performing athletes, and although the Achilles tendon and the patellar tendon are the most commonly affected sites, tendons in, for example, shoulder, foot, or elbow are also affected in sports‐active individuals. Achilles tendinopathy impacts 6% of the general population and up to 50% of all endurance runners over their lifespan [1]. A meta‐analysis on patellar tendinopathy in athletes has recently shown that across sports disciplines, 11% of females and 17% of males had patellar tendinopathy, and that in jumping sports like volleyball and basketball, the prevalence was 20%–35% [2, 3, 4]. Although the pathogenesis of tendinopathy is not fully elucidated, there is less documentation for it being a significant mechanical rupture and rather reflects an overload and disturbed diurnal homeostasis with accompanying swelling, hypervascularization, and gradual morphological changes of the tendon, and accompanying subjective feeling of soreness and pain.

### 1.1 Current Treatment of Tendon Overuse Injury

In an overview regarding all types of treatment for Achilles tendinopathy [5], it has clearly been shown that loading exercise therapies by far surpass non‐loading therapies or wait‐and‐see policy. Currently, muscle strength training of either concentric or eccentric nature (typical over a 12‐week period) combined with a reduction in load during sports is the best evidenced treatment for tendinopathy [6, 7, 8, 9], even when the intensity of the strength training is reduced from 80%–90% 1 RM to 55% 1 RM [10]. In elite athletes, just adding controlled strength training on top of the current training bouts does not have any beneficial effect [11]. It is still an open question as to how much the reduction in regular sports exercise should be along with the recommended treatment with heavy slow resistance training, but limiting sports activity to a self‐monitored pain scale of between 2 and 5 on a 10‐point pain scale has been found not to limit improvement in tendinopathy symptoms over the period with strength training treatment [12]. Some data indicate that improvement in tendinopathy recovery is faster the shorter time the condition has been present. This has been shown in recreational athletes [13], whereas a similar trend was not clear in elite athletes where recovery was independent of whether the condition has been present for 1, 2, or 3 months in the athlete [14].

Platelet‐rich plasma (PRP) has been used in several studies in relation to tendinopathy based on the notion that it contains numerous growth factors that are crucial for the regeneration of tendon tissue. However, well‐controlled studies have shown that there is no significant effect of PRP treatment [15, 16].

Similarly, it has been shown that there is no effect of using anti‐inflammatory medication in the form of oral non‐steroidal anti‐inflammatory drugs (NSAIDs) in both late and early stages of Achilles and patellar tendinopathy [13, 17]. This is a conundrum since there are observations of inflammatory cell accumulation in the early phase of tendinopathy [18]. However, the absence of effect may be because the drug cannot enter the vital areas of the affected tendon [19]. In lateral elbow pain, a minor positive effect of local topical gel administration of NSAID has been documented [20, 21]. Insulin‐like growth factor I is known to be involved in cell proliferation, collagen production, and tissue remodeling. However, local administration of insulin‐like growth factor I into the patellar tendon in combination with strength training in patients with tendinopathy did not improve the treatment outcome beyond than achieved by resistance training alone [22]. Use of local corticosteroids in the peritendinous region to cure tendinopathy has a long history and has repeatedly shown to provide a very good effect, especially in the short term [6, 23, 24]. A recent study used corticosteroids in combination with strength training in Achilles tendinopathy (while abstaining from sports participation) and could demonstrate improved clinical and physiological effects without any adverse result [25]. In contrast, in lateral elbow tendinopathy, there was no improvement with corticosteroid administration in addition to strength training [26]. High‐volume injection treatment involves injecting a large volume of saline, corticosteroid, and anesthetic between the Achilles tendon and Kager's fat pad. It has been shown to have significant beneficial effects upon tendinopathy [27], but the majority of the effect was explained by the corticosteroid added to the injected volume [28]. Shock wave therapy has been shown to have a positive effect upon tendon disorder if there was an intratendinous calcification typical in the shoulder [29], and only minor or no effect was found upon non‐calcified tendinopathy [30]. Laser therapy has, apart from a small pain‐reducing effect, no major treatment effect on tendinopathy [31]. Finally, surgery has been tried in a number of chronic tendinopathic conditions, and on patellar tendinopathy, the effect of surgery has been shown to be equal to heavy resistance training [32] whereas a significant beneficial effect of surgery vs. conservative treatment with training and corticosteroid injection has been shown in chronic fascia plantaris tendinopathy [33].

### 1.2 Unsolved Questions in the Treatment of Tendon Overuse Injury in Athletes

The signs of early development of tendinopathy need to be investigated further to prevent the exacerbation of the disorder, and likewise we need to know more regarding what signs are indicative of when to resume competitive exercise without too much risk of getting a new period of tendinopathy. When using strength training as treatment for tendinopathy, it would be helpful to know what a sufficient amount of controlled resistance exercise necessary for the treatment of tendinopathy is, and maybe even more important, how much sport‐specific training can be allowed during the period of treatment with controlled heavy resistance training. Despite a good treatment effect of heavy resistance training, this does still not cure the condition 100%, and the question remains whether the combination of training with other treatments of pharmacological or physical nature improves the treatment outcome further. To what extent surgery can have any beneficial effect within the treatment of tendinopathy is yet to be documented. Finally, knowing that circadian rhythm is important for tendon homeostasis, it will be interesting to discover whether impaired homeostasis in tendinopathy can be benefited from improved circadian rhythm by applying physical exercises at a specific time point of the day.

## 2 Acute Muscle Strain Injury

Explosive movements or extreme stretches of typically hamstrings or calf muscle can result in an acute rupture of the muscle‐tendon complex with an abrupt discontinuation of athletic movements. Hamstrings muscle injuries are the most common muscle injury in several sports [34, 35, 36]. The most common site for these muscle strain injuries is near the myotendinous junction [37, 38]. Depending upon the severity of the rupture, the athlete is away from sports for weeks to several months [39], and often recurrent injuries at the same primary site (up to 14%–67%) can be observed when training is resumed [40, 41]. The effectiveness of rehabilitating these injuries is of major importance in efforts to avoid re‐injury and for individual athletic performance and thus for success in team sports like football at the highest level [42, 43].

### 2.1 Current Treatment of Acute Muscle Rupture Injury

In randomized controlled trials published on the rehabilitation of acute muscle strain injuries, the time for return to sport/play (RTS/RTP) is from around 2–12 weeks [44, 45, 46, 47, 48, 49, 50, 51]. A review of the literature on the treatment of acute hamstring injuries documented that training rehabilitation with a focus on progressive eccentric strength exercises and gradually increasing running drills yielded the fastest return to play, and at the same time, the lowest re‐injury rate [52]. It has been suggested that early onset of rehabilitation is beneficial [51], but it is debated what exercises and at what time they should be initiated. Lengthening exercises have been suggested to be initiated already 5 days after injury [44, 45], but comparing initiation at 5 days compared to 16 days did not demonstrate any difference in RTS or re‐injury rate [50]. This was found despite improved eccentric strength in the group that started earlier [50]. Bayer et al. [51] showed that the strength improvement during the rehabilitation period was similar in early and delayed initiation of rehabilitation, despite the early rehabilitation group resuming their full activity level 3 weeks earlier than the ones starting their rehabilitation just 1 week later.

In a study that evaluated pain during activity, maximal muscle strength, flexibility, and area of palpatory soreness in sports‐active individuals with hamstring muscle strain injury, it was demonstrated that in 1/3 of the recovery period prior to RTS, the length of the area with palpatory soreness was reduced by 50% [53]. This is of clinical importance as it helps predict the overall duration of the injury recovery. Very recently, a comprehensive study of acute muscle strain injuries in the hamstrings demonstrated that two clinical tests (lower straight leg rise angle on the injured leg and discomfort during active knee extension test), two MRI characteristics (involvement of the MTJ region and low degree of anteroposterior edema), and shorter time to RTS/RTP were all independently associated with increased re‐injury risk for hamstrings [54]. The discomfort during knee extension tripled the risk for re‐injury, as did (×3) the demonstration of affected MTJ region with MRI. Suboptimal straight leg test only marginally increased the risk for re‐injury, and edema observed on the MRI was, in fact, inversely related, that is, larger edema was associated with a smaller risk of re‐injury. Further, it was found that a longer period of RTS/RTP decreased the risk for re‐injury with ~1.5% per day, meaning that a prolonged rehabilitation of 1 week (7 days) potentially would decrease the risk for re‐injury by around 10%, which is clinically very relevant [54]. This raises the question as to what parameters should be monitored before returning to especially elite sports with high demands. It further underlines that injuries located in the myotendinous junction are severe and require a long time before full function can be resumed.

Interestingly, it has been shown that muscle atrophy of the ruptured muscle remains up to 6 months post‐injury, along with increased blood flow in the region [55]. Further, even several years after the injury, abnormal connective tissue, fat accumulation, and hypervascularization can be observed in the injured region [56]. Although strength training at a late stage after muscle strain injury can partly improve symptoms and function, it is mostly the agonist muscles that are improved and the abnormal tissue composition in previously injured muscle persist [56]. Moreover, long after injury, the injured muscle appears disorganized and has a thicker aponeurosis [57]. This indicates lasting tissue morphology changes that may be irreversible changes in the muscle–tendon unit after acute muscle strain injury.

It has been demonstrated that the use of eccentric strengthening exercises (as, e.g., Nordic hamstrings exercise) can not only reduce the risk of primary hamstring injury but will also markedly lower the risk for re‐injury [58, 59, 60]. Further, it might be that a combination of different eccentric exercises even protects the hamstring muscle further against reinjuries [61], but curiously the overall number of hamstring injuries in elite football has not dramatically diminished. This may be due to low compliance and adaptation of the exercises needed in the daily training practices [59]. Other supplementary treatments after muscle strain injury have been tried, such as the administration of PRP into the region of muscle injury, which did not affect RTS or frequency of re‐injury [62, 63]. Recently, the administration of protein supplementation daily for 12 weeks has been tried in individuals with calf or hamstring muscle injury, but this did also not influence RTS (Mertz unpublished observation).

### 2.2 Unsolved Questions in Treatment of Acute Muscle Strain Injury in Athletes

Several clinical descriptions of the rehabilitation process have been provided, but we have little knowledge of the exact healing processes in the injured area and how we can best stimulate such a healing process to achieve an optimal outcome. Further, it is unknown to what degree the regenerating injured tissue will fully resolve after rehabilitation and when the athlete is ready to return to sports at a high level with the lowest possible risk for re‐injury. Moreover, how to evaluate the “readiness” of the tissue in relation to injury rehabilitation remains unknown and if treatments other than mechanical loading (strength training, gradual onset running) can improve the healing process (e.g., inhibition of inflammation) and thus accelerate the time to become ready for sports again.

## 3 Tendon Rupture Injury

Acute Achilles tendon ruptures are the most common tendon rupture in recreational and competitive athletes with a male‐to‐female ratio of 3:1 [64, 65, 66, 67, 68]. Patient‐reported outcome measures (PROMs) show that sports‐active individuals only reach 73–82 out of 100 points even several years after the injury, which indicates that functional recovery after tendon rupture is incomplete [20, 21]. In fact, patients are often left with permanent tendon lengthening, muscle atrophy, and weakness [69], which can result in a reduced performance level or a complete inability to return to the sport at all, that is, devastating consequences for the professional athlete [66, 70, 71, 72].

### 3.1 Current Treatment of Tendon Rupture Injury

A long‐standing dilemma regarding the treatment of, for example, Achilles tendon rupture has been whether to perform surgical suturing of the tendon or to treat the ruptured tendon conservatively with immobilization in a fixed plantarflexion position [73, 74, 75, 76]. A recent large randomized controlled trial found that surgery did not result in better patient‐reported outcomes compared to nonoperative treatment, but it should be noted that patients (which were recreational athletes as well as regular non‐trained patients) were left with a permanent 20%–30% functional deficit irrespective of the treatment approach [76]. Importantly, both surgical and non‐surgical treatments result in tendon elongation, although the non‐surgical approach seems to result in an even longer tendon [77]. Importantly, the elongated tendon has been correlated to reduced physical function [78, 79]. These findings speak in favor of elite athletes who want to return to high‐level sports potentially benefiting more from operative rather than non‐operative treatment.

Much of the existing literature on tendon length after an Achilles tendon rupture has been focused on that associated with the gastrocnemius. The free Achilles tendon is comprised of distinct tendons arising from the three triceps surae muscles [80], but prior studies of Achilles tendon rupture have only considered the length of the gastrocnemius tendon and not that of the soleus tendon. Precise measures of these distinct tendon portions are difficult, but a 3D MRI approach makes it possible to accurately assess the length [79]. Using this technique, it has been shown that there is a marked difference between the injured and uninjured soleus tendon lengths, but not the gastrocnemius tendon 1 week after surgery [81]. This suggests that the current surgical approach may not be optimal for avoiding the prolongations of the different tendons.

It has been shown that 6 weeks after surgery the tendon is elongated up to 10%–20% of the entire length of the free tendon, and in some cases as much as 50% [82]. However, it appears that the tendon continues to elongate up to 6 months after surgery, and that only 50% of the elongation occurs in the initial 3 months [83]. Importantly, the clinical outcome is inversely related to the magnitude of tendon elongation [78, 79, 84, 85]. Delayed loading after surgical repair does not impact tendon elongation or muscle function, which does not recover completely by 12 months [81]. Interestingly, the delayed rehabilitation yielded reduced tendon inflammation in the first 3 months and a greater and meaningful improvement in the **patient‐reported outcome** after 1 year compared to the conventional early rehabilitation [81].

Despite extensive rehabilitation efforts, muscle weakness can persist for years and even decades, which has been attributed to a reduction in anatomical muscle cross‐sectional area [77, 86, 87]. This has prompted a focus on early mobilization following the repair of the ruptured tendon to avoid muscle atrophy [88]. Yet, these efforts have proven ineffective, which is noteworthy since the triceps surae muscles can lose 30% of their mass during a 90 days bed rest and fully regain it in the following 90 days [89]. Therefore, immobilization‐associated anatomical cross‐sectional muscle atrophy, per se, is an unlikely mechanism for the permanent loss of muscle mass and excursion after Achilles tendon rupture.

Impaired range of motion during a heel rise has been correlated to tendon elongation after an Achilles tendon rupture, which appears to be associated with a shorter muscle [79]. The number of parallel sarcomeres governs the force‐generating capacity, while the number of sarcomeres in series governs the muscle's total excursion, i.e., joint range of motion [90]. If the tendon is elongated and the muscle is consequently shortened, the force‐generating capacity could theoretically be maintained if the serial sarcomere number adjusts to maintain the optimal actin‐myosin overlap. However, the shorter muscle would generate less force closer to the maximal plantarflexion angle [91], which is typically seen clinically [81, 91, 92], Recently, it was shown that Achilles tendon elongation results in a loss of muscle mass and length with an accompanying normalization in serial sarcomere count in an animal model [93]. It is possible that the tendon elongation that is accompanied by a shortening of the muscle can explain the reduced end‐range muscle strength and heel‐rise height.

### 3.2 Unsolved Questions in the Treatment of Tendon Rupture in Athletes

Although there is some evidence that a surgical approach is preferential for elite athletes, the optimal treatment remains an enigma, and several questions are unanswered. For example, is there a surgical technique that can restore the anatomical configuration and length of the Achilles tendons' sub‐sections? Further, when is the best time to initiate loading to avoid tendon elongation and muscle shortening, and how should this loading be progressed? Associated with this rehabilitation program, how can we best monitor tendon elongation and tissue inflammation during rehabilitation noninvasively.

## Conflicts of Interest

The authors declare no conflicts of interest.

## Data Availability

This is a narrative review.
